# Development and Evaluation of an Improved Apparatus for Measuring the Emissivity at High Temperatures

**DOI:** 10.3390/s21186252

**Published:** 2021-09-17

**Authors:** Mariacarla Arduini, Jochen Manara, Thomas Stark, Hans-Peter Ebert, Jürgen Hartmann

**Affiliations:** 1 Bavarian Center for Applied Energy Research (ZAE Bayern), 97074 Wuerzburg, Germany; jochen.manara@zae-bayern.de (J.M.); thomas.stark@zae-bayern.de (T.S.); hans-peter.ebert@zae-bayern.de (H.-P.E.); juergen.hartmann@fhws.de (J.H.); 2 Institute Digital Engineering (IDEE), University of Applied Science Wuerzburg-Schweinfurt (FHWS), 97421 Schweinfurt, Germany

**Keywords:** emissivity, reflectivity, infrared radiation, high temperature, FTIR-spectrometer, blackbody, uncertainty, X-point, inductive heating, direct radiative method

## Abstract

An improved apparatus for measuring the spectral directional emissivity in the wavelength range between 1 µm and 20 µm at temperatures up to 2400 K is presented in this paper. As a heating unit an inductor is used to warm up the specimen, as well as the blackbody reference to the specified temperatures. The heating unit is placed in a double-walled vacuum vessel. A defined temperature, as well as a homogenous temperature distribution of the whole surrounding is ensured by a heat transfer fluid flowing through the gap of the double-walled vessel. Additionally, the surrounding is coated with a high-emitting paint and serves as blackbody-like surrounding to ensure defined boundary conditions. For measuring the spectral directional emissivity at different emission angles, a movable mirror is installed in front of the specimen, which can be adjusted by a rotatable arrangement guiding the emitted radiation into the attached FTIR-spectrometer. The setup of the emissivity measurement apparatus (EMMA) and the measurement procedure are introduced, and the derived measurement results are presented. For evaluating the apparatus, measurements were performed on different materials. The determined emissivities agree well with values published in literature within the derived relative uncertainties below 4% for most wavelengths.

## 1. Introduction

Within the EU-funded project Hi-TRACE with the title “industrial process optimization through improved metrological methods for the determination of thermophysical properties” new techniques will be developed for characterizing the thermophysical properties of materials at high temperatures [1]. This includes the design of metrological infrastructure for performing traceable measurements of the temperature of fusion *T*_f_, the thermal contact resistance *R*_c_, the thermal diffusivity *a*, the specific heat capacity *c*_p_ and the emissivity *ε* at high temperatures, which leads to the acronym Hi-TRACε or Hi-TRACE.

This paper focuses on the improvement of an apparatus for measuring the emissivity. Especially at high temperatures, the influence of the radiative heat transfer dominates as the emitted radiation increases with the fourth power of temperature, provided the emissivity is constant with temperature. Hence, in numerous fields, such as aerospace, power plant technology, as well as glass and ceramics productions, the exact knowledge of the emissivity of the deployed materials and components at their operation temperature is of essential importance. However, the emissivity of a surface strongly depends not only on the material but also on the morphology of the material, as well as on the roughness and oxidation state of the surface. Literature data are often only of limited reliability because not all relevant material and surface properties are given with the listed values. Therefore, the emissivity of each certain specimen needs to be determined separately under operational conditions using a reliable, accurate and validated measurement method. In general, there are two methods for determining the emissivity [2]:

- Calorimetric method: the calorimetric method can be used to determine the total hemispherical emissivity $\varepsilon_{h}$ of a surface by measuring the total radiative heat flux emitted from that surface [3,4].
- Radiometric method [5]: the radiometric method is used to determine the spectral directional emissivity $\varepsilon_{d,\lambda }$ (direct radiometric method [6]), or the spectral directional-hemispherical reflectivity $\rho_{\mathrm{dh},\lambda }$ or spectral directional-directional reflectivity $\rho_{\mathrm{dd},\lambda }$ (indirect radiometric method [7]).

This work deals with the direct radiometric method for performing measurements at high temperatures, which is technically more complex than the calorimetric method, but provides more information, especially on the spectral and angular dependence of the emissivity. Several working groups have already performed direct radiometric measurements, which are described, for example, in Refs. [8,9,10,11,12,13,14,15,16,17,18,19,20,21].

A setup for determining the spectral directional emissivity at elevated temperatures has also been developed at the Bavarian Center of Applied Energy Research (ZAE Bayern) [22] and extended previously at ZAE Bayern [23]. Additionally, another setup is available at ZAE Bayern, namely the integrating sphere (IS) setup, which is an indirect radiometric method [24]. With this indirect radiometric method, the spectral directional-hemispherical reflectivity and transmissivity can be determined at ambient temperature and the spectral directional emissivity at ambient temperature can be derived subsequently.

The emissivity measurement apparatus (EMMA) at ZAE Bayern has been significantly improved by applying new components for increasing the reliability and the temperature range up to 2400 K. These new components are mainly a novel vacuum vessel, an optimized beam path with movable mirrors and a new heating unit, which enables inductive heating for reaching very high temperatures. The improved setup is presented hereafter together with the detailed data analysis. Furthermore, selected results are presented and compared with available data from literature.

## 2. Theory and Measurement Procedure

### 2.1. Spectral Directional and Total Directional Emissivity

The measurement procedure of the EMMA and the underlying theory was described in detail in a previous publication [22]. Hence, only a brief introduction for measuring opaque specimens with vanishing transmissivity is given below.

The measured spectral directional radiative intensity $i_{\lambda ,\mathrm{meas}}$ of a specimen depends on the spectral directional emissivity $\varepsilon_{d,\lambda }$, the spectral hemispherical-directional reflectivity $\rho_{\mathrm{hd},\lambda }$, the specimen temperature *T*_sp_ and the temperature of the black surrounding *T*_amb_ [22]

(1) $$i_{\lambda ,\mathrm{meas}}\left(\theta ,T_{\mathrm{sp}},T_{\mathrm{amb}}\right)=\varepsilon_{d,\lambda }\left(\theta ,T_{\mathrm{sp}}\right)\cdot i_{\lambda ,\mathrm{bb}}\left(T_{\mathrm{sp}}\right)+\rho_{\mathrm{hd},\lambda }\left(\theta ,T_{\mathrm{sp}}\right)\cdot i_{\lambda ,\mathrm{bb}}\left(T_{\mathrm{amb}}\right)\;.$$

$i_{\lambda ,\mathrm{bb}}$ gives the spectral directional radiative intensity of a blackbody and *θ* the emission angle. The first term in Equation (1) represents the intensity emitted by the specimen itself and the second term represents the part of the intensity coming from the hemispherical surrounding, which is reflected by the specimen into the view of observation.

Due to the law of reciprocity ($\rho_{\mathrm{dh}}=\rho_{\mathrm{hd}}$), conservation of energy for nontransparent specimen ($\alpha_{d,\lambda }+\rho_{\mathrm{dh}}=1$) and Kirchhoff’s law, which describes the identity of spectral directional absorptivity and emissivity ($\alpha_{d,\lambda }=\varepsilon_{d,\lambda }$), Equation (1) can be solved for the spectral directional emissivity [22]

(2) $$\varepsilon_{d,\lambda }\left(\theta ,T_{\mathrm{sp}}\right)=\frac{i_{\lambda ,\mathrm{meas}}\left(\theta ,T_{\mathrm{sp}},T_{\mathrm{amb}}\right)-i_{\lambda ,\mathrm{bb}}\left(T_{\mathrm{amb}}\right)}{i_{\lambda ,\mathrm{bb}}\left(T_{\mathrm{sp}}\right)-i_{\lambda ,\mathrm{bb}}\left(T_{\mathrm{amb}}\right)}\;.\;$$

Hence, the radiation coming from the black surrounding influences the measurement and has to be considered for the analysis of the derived data. Therefore, the existence of a black surrounding with a constant and homogeneous temperature is inevitable for measuring the emissivity by a direct radiometric method.

Finally, the total directional emissivity $\varepsilon_{d}$ can be calculated from the spectral directional emissivity $\varepsilon_{d,\lambda }$ using Equation (3) [22]

(3) $$\varepsilon_{d}\left(\theta ,T_{\mathrm{sp}}\right)=\frac{\int_{0}^{\infty }\varepsilon_{d,\lambda }\left(\theta ,T_{\mathrm{sp}}\right)\cdot i_{\lambda ,\mathrm{bb}}\left(T_{\mathrm{sp}}\right)\cdot d\lambda }{\int_{0}^{\infty }i_{\lambda ,\mathrm{bb}}\left(T_{\mathrm{sp}}\right)\cdot d\lambda }\;.$$

**Figure 1:**
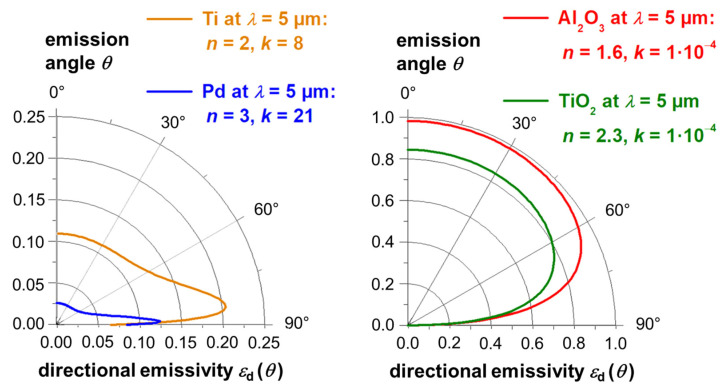
Directional emissivity at ambient temperature as a function of the emission angle between 0° to 90° (relative to the surface normal) for electrically conducting materials (on the left side) and electrically non-conducting materials (on the right side). The calculations have been performed for selected materials with the respective refractive indices taken from [26], assuming opaque specimen with sufficed thicknesses.

Usually, the directional emissivity of electrically non-conducting materials decreases with increasing emission angle whereas the directional emissivity of electrically conducting materials increases with increasing emissions angle. For visualization, the directional emissivity is shown in Figure 1 for different values of the complex refractive index *m* = *n* +*I*·*k* (with the real part *n* and the complex part *k* of the refractive index), which are typical for electrically conducting materials (left side of Figure 1) or electrically non-conducting materials (right side of Figure 1). The calculations have been performed using the formula given in [25] for metals and dielectric materials (with non-vanishing, but very small values of *k*).

Besides the angular dependence it can be seen, that electrically conducting materials usually have emissivities below ca. 0.5, whereas electrically non-conducting materials exhibit emissivities above ca. 0.5 in the infrared spectral region at most emission angles.

### 2.2. Calibration of the Emissivity Measurement Apparatus

Prior to performing measurements with the EMMA, the apparatus has to be calibrated. For this purpose, the measured intensity $i_{\lambda ,\mathrm{meas}}$, which has been introduced in Equation (1), has to be derived from the so-called detected intensity $i_{\lambda ,\mathrm{detected}}$, which is the signal received by the detector. $i_{\lambda ,\mathrm{detected}}$ is influenced by additional factors, as explained previously in Ref. [23]. Referring to the detailed derivation in Ref. [23] one obtains the measured intensity $i_{\lambda ,\mathrm{meas}}$ from the detected intensity $i_{\lambda ,\mathrm{detected}}$ by the following equation

(4) $$i_{\lambda ,\mathrm{meas}}=\frac{i_{\lambda ,\mathrm{detected}}-C_{2}\left(\lambda \right)}{C_{1}\left(\lambda \right)}\;.\;$$

**Figure 2:**
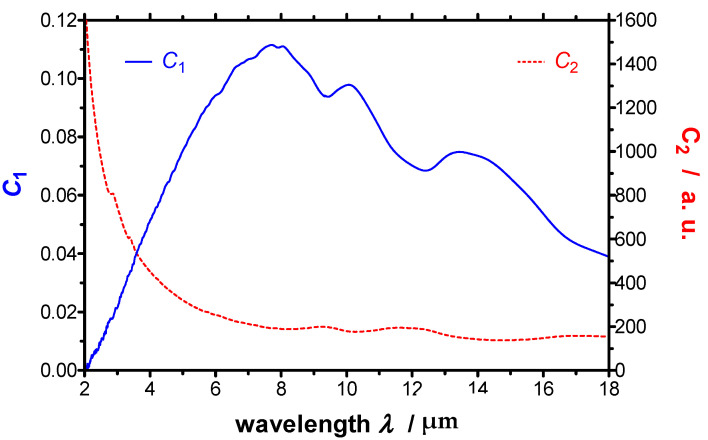
Typical calibrating functions *C*_1_ and *C*_2_ derived from a calibration of the apparatus versus wavelength.

**Figure 3:**
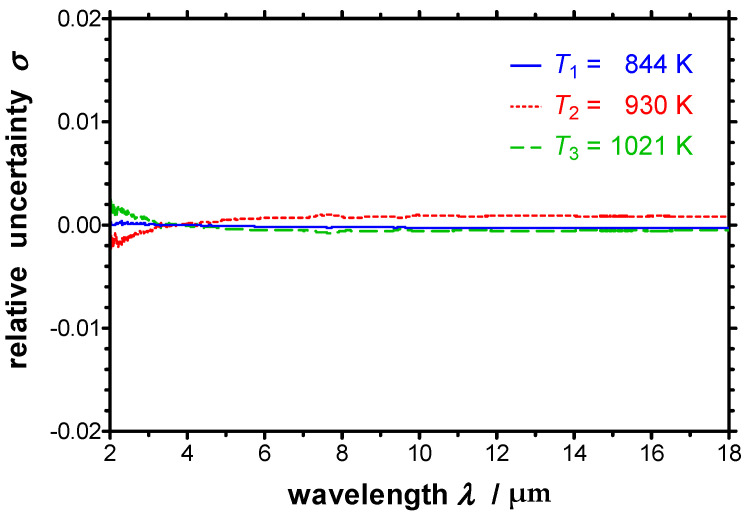
Spectral relative uncertainty of the calibration of the improved EMMA for three calibration temperatures *T*_1_, *T*_2_ and *T*_3_.

$C_{1}\left(\lambda \right)$ quantifies the attenuation of the emitted radiation by mirrors, apertures, etc., whereas $C_{2}\left(\lambda \right)$ quantifies the gain of detected radiation due to thermal radiation, which is emitted from spectrometer components at near-ambient temperature [27]. Exemplarily, both calibrating functions $C_{1}\left(\lambda \right)$ and $C_{2}\left(\lambda \right)$ are depicted in Figure 2 as function of the wavelength derived from test measurements. The calibration procedure and the derivation of the calibrating functions $C_{1}\left(\lambda \right)$ and $C_{2}\left(\lambda \right)$ from the calibration measurements on a reference blackbody at three different temperatures are described in detail in Ref. [23]. The resulting relative uncertainty *σ* at these three temperatures are given in Figure 3 as function of the wavelength. It can be seen that the relative uncertainty due to the calibration lies below 0.2% and is therefore only of minor relevance.

## 3. Experimental Details

### 3.1. Configuration of the Emissivity Measurement Apparatus

**Figure 4:**
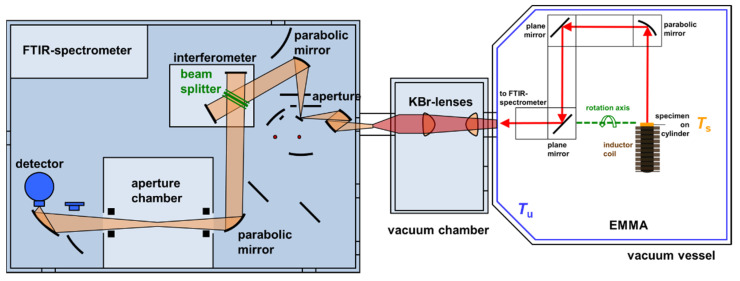
Sketch of the improved EMMA. The inductor is placed in the vacuum vessel on the right, which is coupled to the FTIR-spectrometer Vertex 70v on the left by an additional vacuum chamber in the middle. The intensity emitted by the specimen is detected by the spectrometer. The whole beam path can be evacuated.

**Figure 5:**
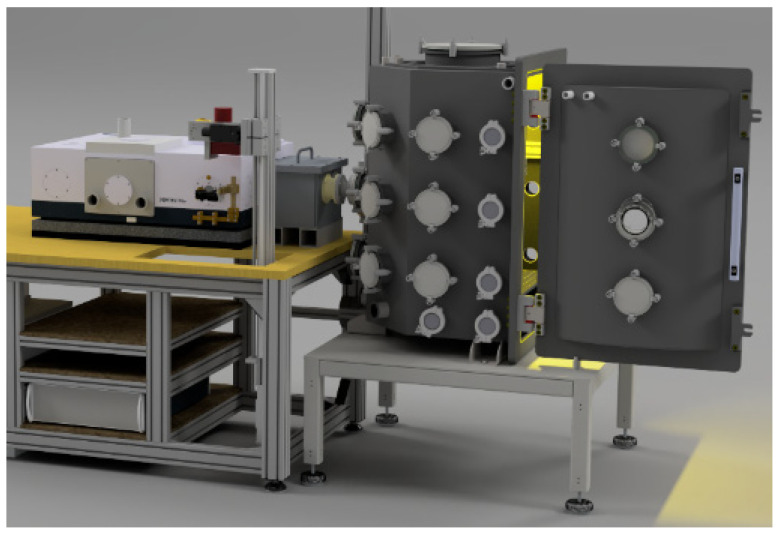
Improved EMMA. The vacuum vessel with the inductive heating unit on the right is attached to the FTIR-spectrometer on the left via the vacuum chamber in the middle.

The improved emissivity measurement apparatus (EMMA), which has been build up with novel customized components, provides an enhanced performance, especially higher accessible temperatures due to an inductive heating unit. Additionally, a new vacuum vessel and an optimized beam path with movable mirrors have been installed. A detailed description of the improved EMMA is given below, whereas a sketch and a photorealistic drawing are depicted in Figure 4 and Figure 5, respectively.

**Figure 6:**
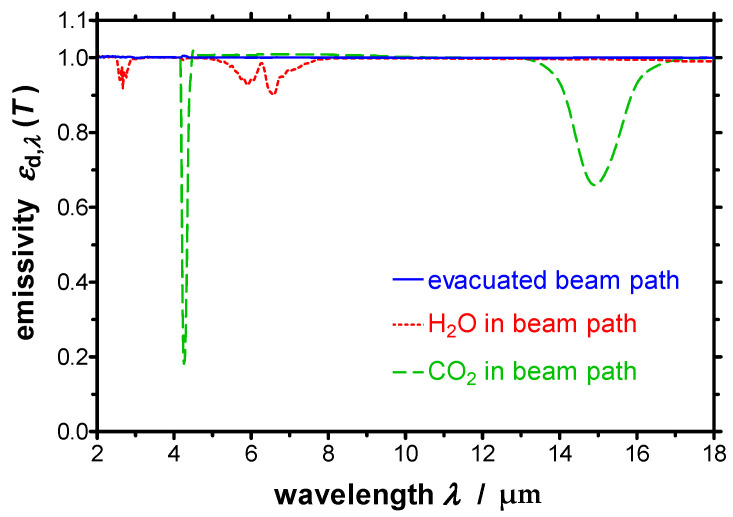
Three measurements of the spectral directional emissivity were realized with a reference blackbody at *T* = 873 K. For the first measurement the beam path was evacuated, then either the H_2_O or the CO_2_ content in the beam path was consecutively increased.

The developed apparatus consists of a double-walled stainless-steel vessel with the internal dimensions (600 × 600 × 900) mm^3^. Working in vacuum eliminates the influence of infrared-active gases such as water vapor or carbon dioxide (see Figure 6) and avoids oxidation of the specimen. The temperature of the double walls of the vessel can be tempered very homogeneously with a stability of ±0.5 K using a heat transfer fluid and a thermostat. This provides a constant and homogeneous temperature of the surrounding, which is essential for deriving the spectral directional emissivity according to Equation (2). A black paint, the Nextel-Velvet-Coating 811-21, serves as high emitting coating which is applied on the interior of the vacuum vessel. This coating exhibits a spectral emissivity of 0.975 ± 0.010 in the relevant infrared spectral region [28].

**Figure 7:**
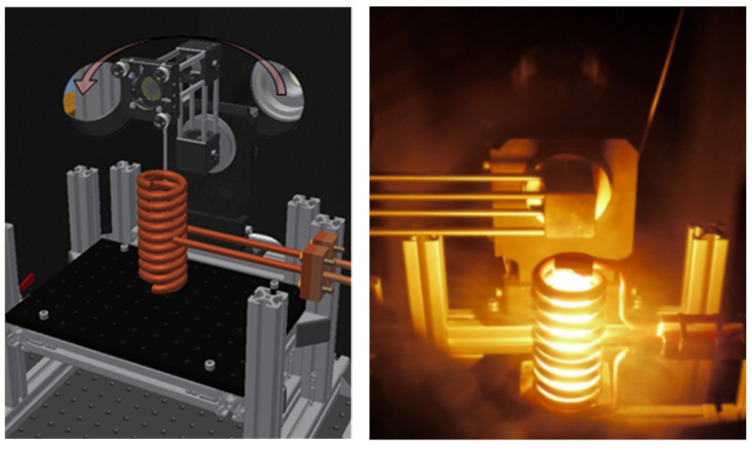
Scheme of the setup of the inductor inside the vacuum vessel with movable mirror arm on the left and photo of inductor with heated reference blackbody on the right.

**Figure 8:**
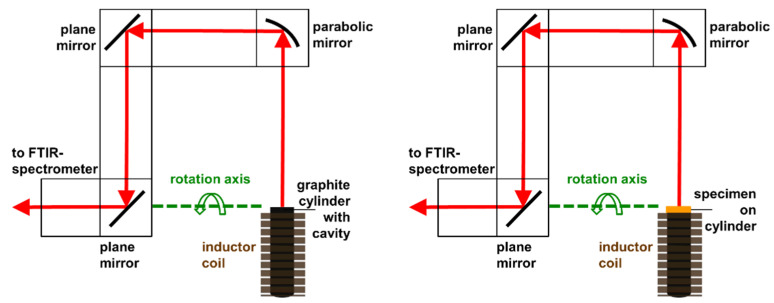
Sketch of the inductor with the movable mirror arm. Configuration for the calibration of the FTIR-spectrometer with a graphite cylinder, which serves as blackbody on the left. Configuration for measuring the emissivity of a specimen, which is heated from the backside, on the right.

The inductor, which is used for heating the specimen and the blackbody, respectively, consists of a copper coil, which is internally water-cooled (see Figure 7). A high frequency alternating current induces eddy currents either inside the specimen itself or inside a supporting graphite cylinder on which the specimen can be placed. The graphite cylinder (20 mm diameter and 100 mm length) with a conic cavity is placed inside the inductor coil. Therefore, the graphite cylinder serves as a blackbody, too. The walls of the cavity exhibit an emissivity significantly above 0.5. This leads to an emissivity of the cavity larger than 0.99 [29]. At first, the apparatus can be calibrated using the described graphite cylinder, which is heated to the desired temperature (see left sketch in Figure 8). Afterwards the emissivity of a specimen can be measured by closing the graphite cylinder with a cap and positioning the specimen on the cap (see right sketch in Figure 8). Depending on the material of the specimen, the specimen can then be heated either directly by induction or indirectly from the backside, where the hot graphite cylinder is located. The radiation coming from the blackbody or the specimen, respectively, is guided by different mirrors to the FTIR-spectrometer and is finally detected by the integrated IR-detector. For this purpose, the vacuum vessel is coupled to a Bruker FTIR-spectrometer Vertex 70v as shown in Figure 4. The three mirrors in Figure 8 are mounted on a moveable mirror arm. By tilting the mirror arm around the rotation axis, it is possible to measure the spectral directional emissivity at different angles up to 90° (relative to the surface normal).

There are several advantages of the improved EMMA in comparison with the previous ones: mainly the inductor for extending the temperature range, but also the completely automatically rotating mirror arm for enhancing the angular dependent measurements and the optimized cooling system of the vacuum vessel for ensuring constant and homogenous surrounding temperatures.

### 3.2. Temperature Measurement

The radiation emitted by a surface of an opaque specimen depends on the emissivity and the temperature of the specimen according to Planck’s law. For determining one of the two parameters, the other one usually has to be known. Hence, the knowledge of the respective temperature is of essential importance for determining the emissivity of surfaces and the resulting accuracy is substantially affected by the accuracies of the determined temperatures.

A contact thermometer (platinum resistance thermometer Pt100) was used to determine the temperature of the coated inner wall of the vacuum vessel, whereas the temperature of the blackbody was measured by a radiation thermometer (Sensortherm Metis MY84 for temperatures below 700 K or Sensortherm Metis M316 for temperatures above 700 K), which is additionally used to control the heating power. The adjustment of the heating power enables a continuous variation of the heating rates and holding times at certain temperatures. The power of the inductor is configured for 5 kW, which ensures fast heating rates if needed.

**Figure 9:**
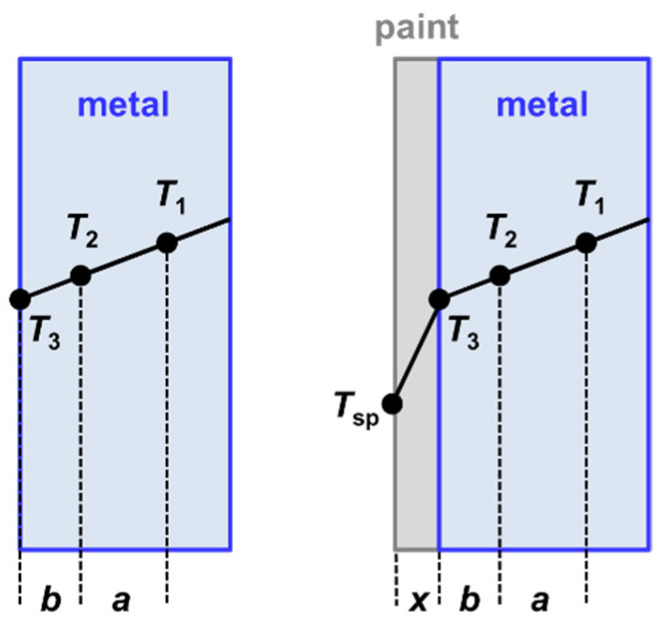
Determination of the surface temperatures of two different specimens by extrapolation, namely a metal (shown on the left) and a paint, which is applied on a metal substrate (shown on the right). *T*_1_ and *T*_2_ are the temperatures, which are detected by two radiation thermometers. *T*_3_ and *T*_sp_ are calculated subsequently.

The measurement of the surface temperatures of the specimens depends on the materials, which are investigated. For oxide ceramics, the so-called Christiansen wavelength can be used for determining the surface temperature, without using further temperature sensors, as described in [30]. To extrapolate the surface temperature of other types of materials from the temperature gradient inside the specimen, usually two holes been prepared in the specimen. For measuring the temperature at the position of the holes, radiation thermometers were focused on the cavities. Figure 9 shows a cross section of two different types of specimens with and without coating together with the locations of the holes. The distance between both positions is *a* = 2 mm and the distance between one position and the interface is *b* = 1.5 mm. A copper specimen serves as a specimen with low emissivity, whereas a black paint (Nextel Velvet 811-21), which is applied on an aluminum substrate, serves as a specimen with high emissivity. Furthermore, a tungsten specimen, which is a refractory metal, serves as high-temperature resistant material.

For thin specimens (thickness << diameter), one dimensional heat transfer dominates and Fourier’s law simplifies to [23]

(5) $$\dot{q}=-\lambda_{\mathrm{material}}\cdot \frac{\partial T}{\partial x}\;.\;$$

$\dot{q}$ gives the local heat flux and *λ*_material_ the thermal conductivity of the material. During the measurement, a stationary temperature gradient is achieved for each surface temperature, at which the emissivity has to be determined. For the metal specimen or the metal substrate (Figure 9), respectively, the surface temperature is calculated as follows:

(6) $$\dot{q}=\lambda_{\mathrm{material}}\cdot \frac{T_{1}-T_{2}}{a}=\lambda_{\mathrm{material}}\cdot \frac{T_{2}-T_{3}}{b}\;\Rightarrow \;T_{3}=T_{2}-\frac{b\cdot \left(T_{1}-T_{2}\right)}{a}=T_{2}-\frac{1.5\;\mathrm{mm}\cdot 0.01\;K}{2.0\;\mathrm{mm}}=T_{2}-0.0075\;K\approx T_{2}\;.\;$$

The measured difference between the temperatures *T*_1_ and *T*_2_ is very small and lower than 0.01 K (i.e., *T*_1_ − *T*_2_ < 0.01 K). The small discrepancies of the temperatures *T*_1_, *T*_2_ and *T*_3_ are due to the high thermal conductivities of aluminum and copper, which are above 200 W/(m·K) and 350 W/(m·K) for temperatures up to 800 K, respectively [31]. Tungsten exhibits also a high thermal conductivity between 175 W/(m·K) at ambient temperature and 93 W/(m·K) at a temperature of 2400 K [32].

The surface temperature of the Nextel-Velvet-Coating 811-21 on an aluminum substrate can be determined analogously

(7) $$\dot{q}=\lambda_{\mathrm{Al}}\cdot \frac{T_{1}-T_{2}}{a}=\lambda_{\mathrm{Nextel}}\cdot \frac{T_{3}-T_{\mathrm{sp}}}{x}\;.\;$$

According to Equation (6) it can be assumed that $T_{3}\approx T_{2}$ and the surface temperature of the paint *T*_sp_ can be determined

(8) $$T_{\mathrm{sp}}=T_{2}-\frac{\lambda_{\mathrm{Al}}}{\lambda_{\mathrm{Nextel}}}\cdot \frac{x\cdot \left(T_{1}-T_{2}\right)}{a}\;.\;$$

The thermal conductivity of the Nextel-Velvet-Coating 811-21 depends slightly on temperature and has a value of *λ*_Nextel_ = 0.192 W/(m·K) at a temperature of 393 K [33]. The thickness *x* of the measured paint was determined by a micrometer gauge with a resulting value of *x* = 110 µm. For this measured thickness and *λ*_Al_ = 220 W/(m·K) [31] one gets $T_{\mathrm{sp}}=T_{2}-0.63\;K$ from Equation (8).

### 3.3. Measurement Accuracy

The standard uncertainty $u_{\varepsilon_{d,\lambda }}$ and the expanded standard uncertainty $U_{\varepsilon_{d,\lambda }}$, respectively, can be derived from Equation (2) using the law of propagation of uncertainties. In this work the expanded standard uncertainty $U_{\varepsilon_{d,\lambda }}=k_{u}\cdot u_{\varepsilon_{d,\lambda }}$ is calculated and discussed for a coverage factor of *k_u_* = 2

(9) $$U_{\varepsilon_{d,\lambda }}\left(i_{\lambda ,\mathrm{meas}},T_{\mathrm{sp}},T_{\mathrm{amb}}\right)=\sqrt{{\left(\frac{\partial \varepsilon_{d,\lambda }}{\partial i_{\lambda ,\mathrm{meas}}}\right)}^{2}\cdot U_{i_{\lambda ,\mathrm{meas}}}^{2}+{\left(\frac{\partial \varepsilon_{d,\lambda }}{\partial T_{\mathrm{sp}}}\right)}^{2}\cdot U_{T_{\mathrm{sp}}}^{2}+{\left(\frac{\partial \varepsilon_{d,\lambda }}{\partial T_{\mathrm{amb}}}\right)}^{2}\cdot U_{T_{\mathrm{amb}}}^{2}},$$

which leads to the following equation for the expanded standard uncertainty of the resulting spectral directional emissivity *ε*_d,*λ*_

(10) $$U_{\varepsilon_{d,\lambda }}(i_{\lambda ,\mathrm{meas}},T_{\mathrm{sp}},T_{\mathrm{amb}})=\frac{\sqrt{\begin{array}{c} {\left[i_{\lambda ,\mathrm{bb}}\left(T_{\mathrm{sp}}\right)-i_{\lambda ,\mathrm{bb}}\left(T_{\mathrm{amb}}\right)\right]}^{2}\cdot U_{I_{\mathrm{meas}}}^{2}\\ +{\left[i_{\lambda ,\mathrm{meas}}\left(T_{\mathrm{sp}},T_{\mathrm{amb}}\right)-i_{\lambda ,\mathrm{bb}}\left(T_{\mathrm{amb}}\right)\right]}^{2}\cdot {\left[\frac{i_{\lambda ,\mathrm{bb}}\left(T_{\mathrm{sp}}\right)}{T_{\mathrm{sp}}^{2}}\cdot \frac{\exp\left(\frac{h\cdot c}{k_{B}\cdot T_{\mathrm{sp}}\cdot \lambda }\right)\cdot \frac{h\cdot c}{k_{B}\cdot \lambda }}{\exp\left(\frac{h\cdot c}{k_{B}\cdot T_{\mathrm{sp}}\cdot \lambda }\right)-1}\right]}^{2}\cdot U_{T_{\mathrm{sp}}}^{2}\\ +{\left[i_{\lambda ,\mathrm{meas}}\left(T_{\mathrm{sp}},T_{\mathrm{amb}}\right)-i_{\lambda ,\mathrm{bb}}\left(T_{\mathrm{sp}}\right)\right]}^{2}\cdot {\left[\frac{i_{\lambda ,\mathrm{bb}}\left(T_{\mathrm{amb}}\right)}{T_{\mathrm{amb}}^{2}}\cdot \frac{\exp\left(\frac{h\cdot c}{k_{B}\cdot T_{\mathrm{amb}}\cdot \lambda }\right)\cdot \frac{h\cdot c}{k_{B}\cdot \lambda }}{\exp\left(\frac{h\cdot c}{k_{B}\cdot T_{\mathrm{amb}}\cdot \lambda }\right)-1}\right]}^{2}\cdot U_{T_{\mathrm{amb}}}^{2} \end{array}}}{\overset{}{{\left[i_{\lambda ,\mathrm{bb}}\left(T_{\mathrm{sp}}\right)-i_{\lambda ,\mathrm{bb}}\left(T_{\mathrm{amb}}\right)\right]}^{2}}}$$

The expanded standard uncertainty of the spectral directional emissivity depends mainly on three components:

- Expanded standard uncertainty of the measured spectral directional intensity $U_{i_{\lambda ,\mathrm{meas}}}$. This uncertainty depends on the uncertainty of the detected signal and the calibration, as well as on the uncertainty of the three different temperatures of the reference blackbody, which are used for the calibration. The relative expanded standard uncertainty of the measured spectral directional intensity is about
  $\frac{U_{i_{\lambda ,\mathrm{meas}}}}{i_{\lambda ,\mathrm{meas}}}=0.015.$
- Expanded standard uncertainty of the measured temperature of the specimen surface $U_{T_{\mathrm{sp}}}$. This uncertainty depends on the uncertainty of the radiation thermometer and the uncertainty of the evaluation procedure. The relative expanded standard uncertainty of the measured temperature of the specimen surface is approximately
  $\frac{U_{T_{\mathrm{sp}}}}{T_{\mathrm{sp}}}=0.015.$
- Expanded standard uncertainty of the measured temperature of the black surrounding $U_{T_{\mathrm{amb}}}$. This uncertainty depends on the uncertainty of the contact thermometer and the homogeneity of the temperature distribution. The relative expanded standard uncertainty of the measured temperature of the black surrounding can be estimated to
  $\frac{U_{T_{\mathrm{amb}}}}{T_{\mathrm{amb}}}=0.010.$

Beside the absolute expanded standard uncertainty of the spectral directional emissivity $U_{\varepsilon_{d,\lambda }}$, the relative expanded standard uncertainty of the spectral directional emissivity $U_{\varepsilon_{d,\lambda }}\cdot \varepsilon_{d,\lambda }^{-1}$ can be determined.

**Figure 10:**
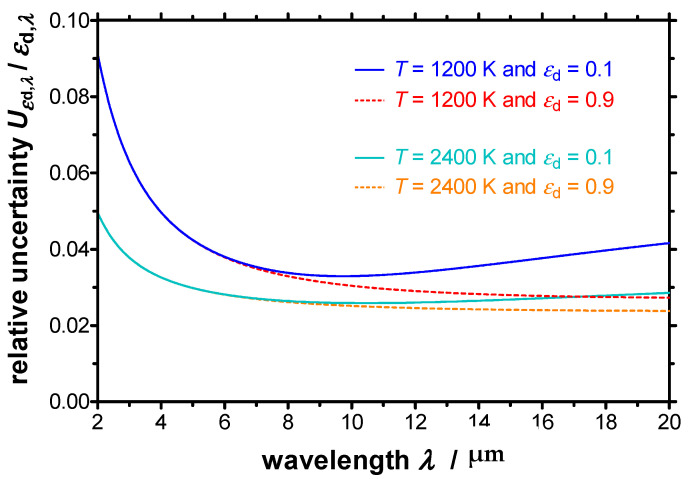
Relative expanded standard uncertainty of the spectral directional emissivity at two different temperatures for a low emissivity of 0.1 and a high emissivity of 0.9.

The relative expanded standard uncertainty of the spectral directional emissivity $U_{\varepsilon_{d,\lambda }}$ has been calculated at two temperatures (*T* = 1200 K and *T* = 2400 K) for a high emissivity of *ε*_d,*λ*_ = 0.9 and a low emissivity of *ε*_d,*λ*_ = 0.1 as a function of wavelength, respectively. The resulting relative uncertainties are depicted in Figure 10. The relative uncertainties lie significantly below 10% for all wavelengths and below 4% for most wavelengths.

In general, the relative uncertainty decreases with increasing temperatures. Furthermore, the relative uncertainty increases for shorter wavelengths below 8 µm, as well as for longer wavelengths above 12 µm for low emissivities. The tendentious course of the relative uncertainties is similar to the characteristics of the relative uncertainties of emissivities measurements, which can be found in the literature [34].

### 3.4. Investigated Specimens

For validating the improved emissivity measurement apparatus (EMMA) three types of specimens were investigated and the results were compared with data from literature. The emissivity was measured at an emission angle of 0° (normal to the surface) as well as at different emission angles up to 70°. A Nextel-Velvet-Coating 811-21 was chosen as an example for a high emitting surface. The Nextel-Velvet-Coating 811-21 was applied on an aluminum disc with a diameter of 20 mm and a thickness of 5 mm. The thickness of the coating was determined to 110 µm. A polished copper specimen (20 mm diameter and 5 mm thickness) with a surface roughness below 0.1 µm was prepared as an example for a low emitting surface. Furthermore, a tungsten specimen (19 mm diameter and 4 mm thickness) was selected as refractory metal, which is temperature resistant up to high temperatures. Prior to the measurements, the tungsten specimen was exposed to a defined heat treatment (2400 K for several hours).

## 4. Results and Discussion

Initially, measurements at temperatures below 1200 K and subsequently at higher temperatures up to 2400 K have been performed in order to evaluate the improved EMMA in the whole temperature region from near-ambient temperature to high temperatures.

### 4.1. Measurement of a High Emtting Specimen

**Figure 11:**
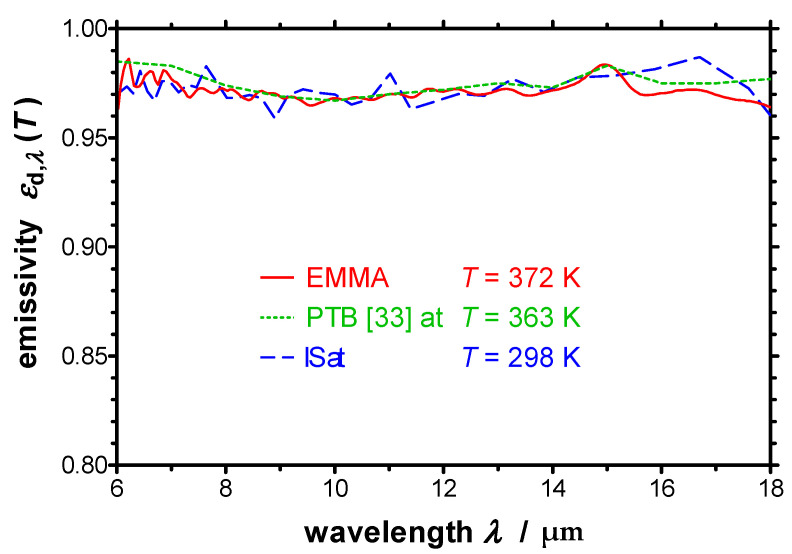
Spectral directional emissivity of a Nextel-Velvet-Coating 811-21 normal to the surface measured with the EMMA at elevated temperature in comparison with the emissivity measured with an integrating sphere (IS) at ambient temperature and with the emissivity measured at PTB [33].

At first, the results of the measurements of the Nextel-Velvet-Coating 811-21 are presented. The spectral directional emissivity *ε*_d,*λ*_ of the paint was measured normal to the specimen surface at different temperatures from 325 K to 420 K. In this temperature range *ε*_d,*λ*_ of the Nextel-Velvet-Coating is almost constant. Figure 11 presents the resulting spectral directional emissivity normal to the surface between 6 µm and 18 µm at *T* = 372 K in comparison with *ε*_d,*λ*_ measured at ambient temperature using an integrating sphere (IS). Additionally, the spectral directional emissivity measured previously at the Physikalisch-Technische Bundesanstalt (PTB) at *T* = 363 K [33] is plotted in Figure 11. Thereby, the values presented in Ref. [33] are in agreement with recent measurements of PTB published in Ref. [35]. One can see that *ε*_d,*λ*_ is almost independent of wavelength and lies around 0.975. Furthermore, the values derived in this work and the values taken from literature are in a good accordance.

**Figure 12:**
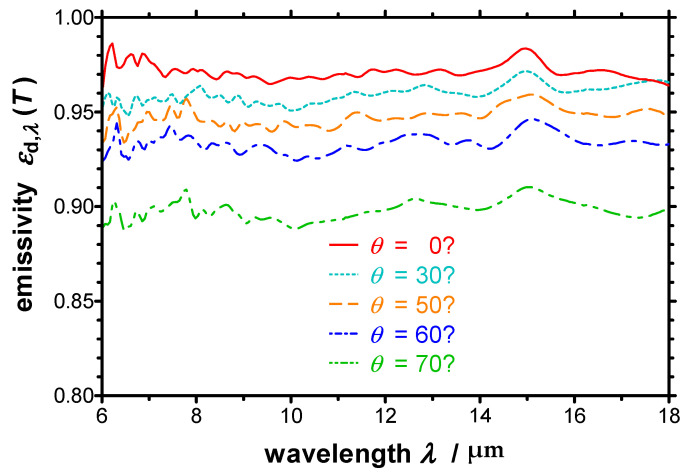
Spectral directional emissivity of a Nextel-Velvet-Coating 811-21 measured with the EMMA at a temperature of *T* = 372 K and different emission angles. The emissivity decreases with increasing emission angle as expected for an electrically non-conducting material.

Besides, the spectral directional emissivity of the Nextel-Velvet-Coating 811-21 was measured at different emission angles from *θ* = 0° (normal to the surface) to *θ* = 70° at a temperature of *T* = 372 K (Figure 12). *ε*_d,*λ*_ decreases with increasing emission angle, as expected for an electrically non-conducting material. The spectral trend of the emissivity is similar for all emission angles.

**Figure 13:**
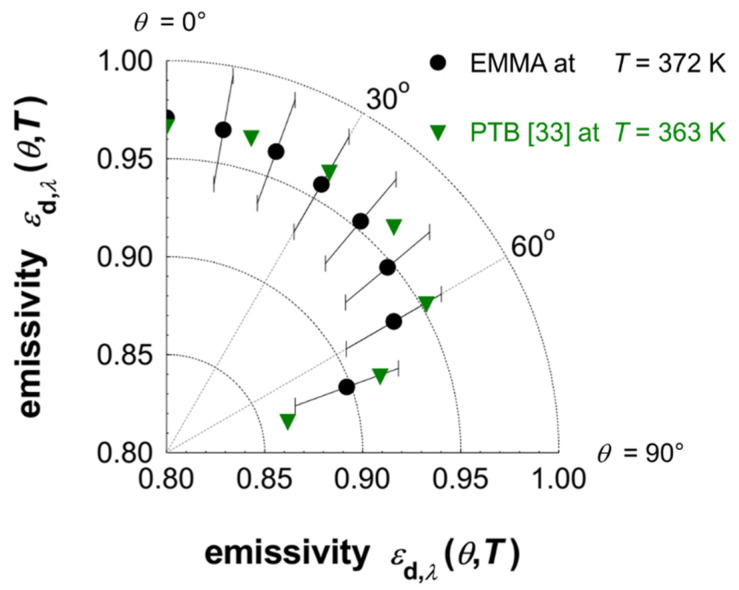
Total directional emissivity of the Nextel-Velvet-Coating 811-21 measured with the EMMA at a temperature of *T* = 372 K and different emission angles in comparison to published data of PTB derived at *T* = 363 K [33]. Additionally, the uncertainty bars are given for the values, which have been derived from the EMMA measurements.

Finally, the total directional emissivity *ε*_d_ at *T* = 372 K was calculated according to Equation (3). The resulting total directional emissivities are shown in a polar diagram (Figure 13) for different emission angles. For comparison the corresponding values derived at PTB at *T* = 363 K [33] are also depicted in Figure 13. There is also a good agreement between the values presented in Figure 13. Only for emission angles above 30° slight differences between the total directional emissivity determined at ZAE Bayern and PTB are visible, which are within the expected uncertainties.

### 4.2. Measurement of a Low Emitting Specimen

Second, a polished copper specimen with a low emissivity was investigated. The configuration of the surface (such as surface roughness and degree of oxidation) drastically influences its emissivity. To ensure a comparison of the measurements performed in this work with published values, the surface of the copper specimen was polished to a roughness lower than 0.1 µm.

**Figure 14:**
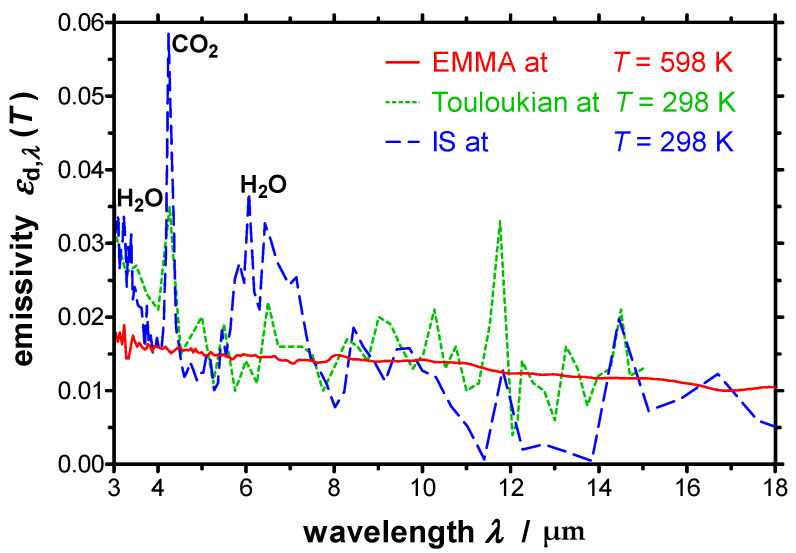
Spectral directional emissivity of polished copper near-normal to the surface measured with the EMMA together with the emissivity measured by an integrating sphere (IS). Additionally, values taken from Ref. [36] are given in the graph for comparison.

Figure 14 reports the spectral directional emissivity near-normal to the surface for wavelengths between 3 µm and 18 µm measured at a temperature of *T* = 598 K together with *ε*_d,*λ*_ measured at ambient temperature using an integrating sphere (IS). Additionally, published data taken from Ref. [36] is given in the graph for comparison. The spectra measured at ambient temperature show a CO_2_-peak at *λ* = 4.3 µm and H_2_O-bonds around 3 µm as well as between *λ* = 5 µm and *λ* = 8 µm, which are not present in the spectrum measured at *T* = 598 K with the EMMA (Figure 14). This is because the integrating sphere is placed outside the evacuated compartment. Again, a good agreement between the values can be seen. *ε*_d,*λ*_ measured at ambient temperature is slightly lower than the one measured at elevated temperature. This is because the emissivity of a metallic surface increases with increasing temperature.

**Figure 15:**
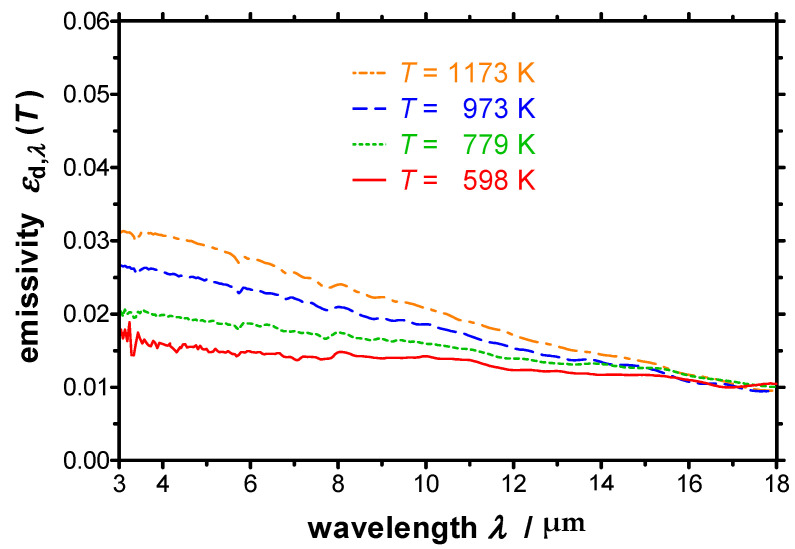
Spectral directional emissivity of polished copper near-normal to the surface measured with the EMMA at different temperatures. The emissivity increases with increasing temperature and with decreasing wavelength.

In Figure 15 the spectral directional emissivity near-normal to the surface is plotted for different temperatures between 2 µm and 18 µm. The increase of the emissivity with increasing temperature is correlated with a decrease of the electrical conductivity with increasing temperature. Furthermore, the spectral emissivity decreases with increasing wavelength, which is correlated with the frequency dependency of the electrical conductivity.

**Figure 16:**
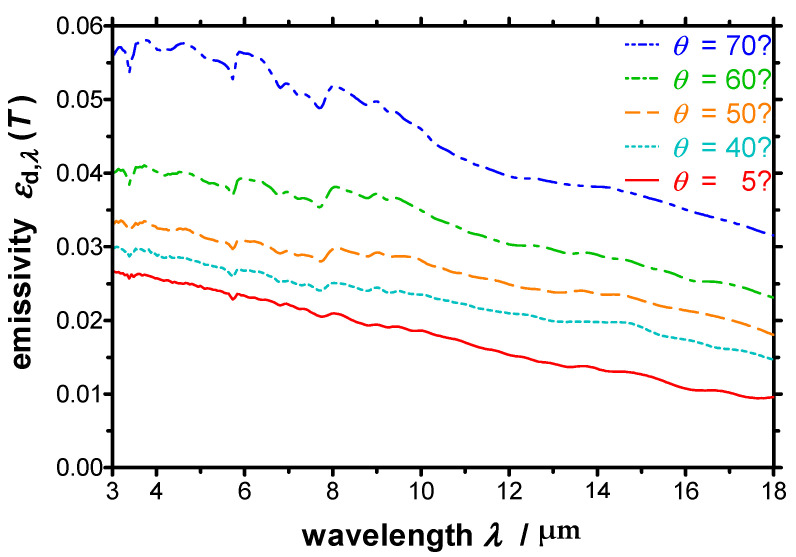
Spectral directional emissivity of polished copper measured with the EMMA at a temperature of *T* = 973 K and different emission angles. The emissivity increases with increasing emission angle as expected for an electrically conducting material.

The spectral directional emissivity of the polished copper specimen measured at a temperature of *T* = 973 K and different emission angles between 5° to 70° (relative to the surface normal) is depicted in Figure 16. The emissivity increases with increasing emission angle, as expected for an electrically conducting material. In this case, the measurement was performed at an angle of 5° instead of 0° to avoid that radiation from the spectrometer or the detector, respectively, and was reflected directly back to the detector by the specular reflecting copper surface. No significant oxidation of the copper specimen occurred as the measurements were performed under vacuum with a pressure below 10^−3^ mbar.

### 4.3. Measurement of a Refractory Metal

**Figure 17:**
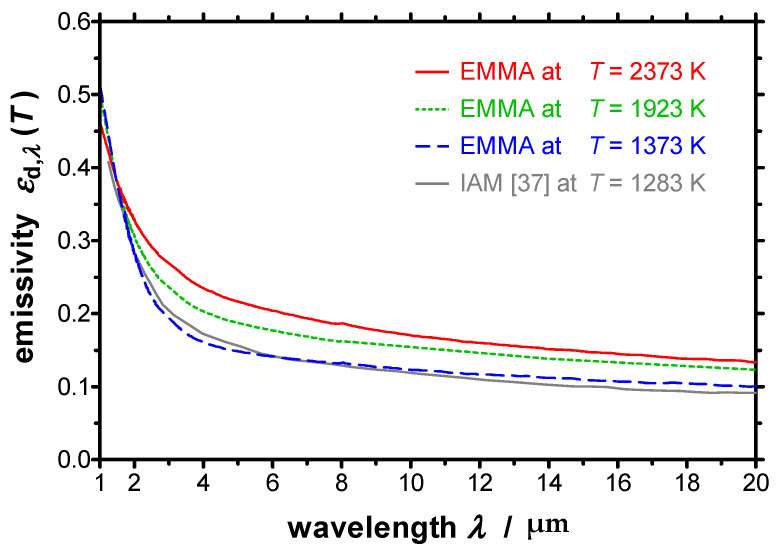
Spectral directional emissivity of tungsten normal to the surface measured with the EMMA at different temperatures between *T* = 1373 K and *T* = 2373 K together with the emissivity measured at IAM at a temperature of *T* = 1283 K [37].

Finally, tungsten, which is a refractory metal, serves as a high-temperature resistant material and has been investigated at temperatures up to *T* = 2373 K. For this purpose, Laboratoire National de Métrologie et d’Essais (LNE) has provided tungsten specimens, which have been further prepared by Physikalisch-Technische Bundesanstalt (PTB). Prior to the measurements reported in this work, the tungsten specimen has been annealed at ZAE Bayern at a temperature of 2400 K for several hours to assure defined properties and reproducible results together with a roughness of about 1 µm. The resulting spectral directional emissivity normal to the surface measured with the EMMA can be found in Figure 17. For comparison a curve derived by the Institut für Angewandte Materialtechnik (IAM) at the University of Duisburg-Essen at a temperature of *T* = 1283 K [37] is additionally plotted in Figure 17. It can be seen that the emissivity derived at IAM is in a good agreement with the emissivity measured with the EMMA at a similar temperature.

**Figure 18:**
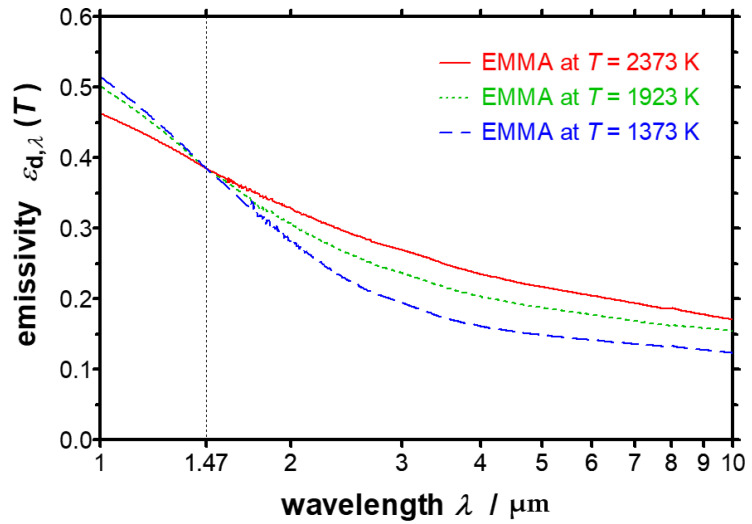
Logarithmic plot of the spectral directional emissivity of tungsten normal to the surface measured with the EMMA at different temperatures between *T* = 1373 K and *T* = 2373 K. The X-point is clearly visible at a wavelength of *λ*_X_ = 1.47 µm.

For a more detailed discussion, a logarithmic plot of the spectral directional emissivity is depicted in Figure 18. It is clearly visible that the spectral curves of the emissivities at different temperatures are all intersecting in one point, the so-called crossover point or X-point. The wavelength of the X-point can be determined to *λ*_X_ = 1.47 µm. For the resulting spectral directional emissivity at the X-point the temperature-independent value $\varepsilon_{d,\lambda_{X}}=0.384$ has been achieved by the EMMA measurements. In Ref. [37] the wavelength and the emissivity at the X-point are given as *λ*_X_ = 1.41 µm and $\varepsilon_{d,\lambda_{X}}=0.380$, which is in a good accordance with the EMMA measurements, too.

Tungsten reveals the typical infrared-optical behavior of metallic surfaces. The emissivity increases with increasing temperature at wavelengths above *λ*_X_. For wavelengths below *λ*_X_ the dependency changes and the spectral emissivity decreases with increasing temperature. This characteristic can be observed for many metals, whereby the position of the X-point varies within the near infrared region depending on the respective material [38].

## 5. Summary and Conclusions

An improved device for determining the spectral directional emissivity at temperatures from near-ambient temperature up to 2400 K, the emissivity measurement apparatus (EMMA) has been developed. Especially the temperature range of an existing setup has been extended by implementing a new inductor for heating up specimens and reference blackbodies, respectively. Furthermore, a double-walled vacuum vessel with a temperature-controlled black surrounding has been installed. This vacuum vessel contains a novel movable mirror arm for performing angular depended measurements of the emissivity for emission angle between 0° and 90° (relative to the surface normal).

Test measurements have been performed on different specimens to evaluate the improved EMMA in the whole temperature range. The results of these measurements are in good agreement with data from literature as the discrepancies are below the expanded standard uncertainties given above. Relative uncertainties below 3% can be reached at a temperature of 2400 K and at wavelengths above 5 µm. At lower temperatures and shorter wavelengths, the relative uncertainty increases, but remains below 4% at a temperature of 1200 K at most wavelengths and significantly below 10% at all wavelengths. Thus, measurements of the spectral directional emissivity can be done with defined accuracies at high temperatures using the EMMA.

In future, the apparatus will be further improved in the EU-funded project Hi-TRACE to reach even higher temperatures up to the temperature of fusion of the investigated materials or up to 3300 K, respectively, which will be supported by round robin tests. Moreover, the accuracy will be further improved, especially at shorter wavelengths.

## Data Availability

Data supporting the reported results are available on request from the corresponding authors.
